# Remote ischemic preconditioning for cardioprotection in elective inpatient abdominal surgery – a randomized controlled trial

**DOI:** 10.1186/s12871-018-0524-6

**Published:** 2018-06-26

**Authors:** Stefan Samad Antonowicz, Davina Cavallaro, Nicola Jacques, Abby Brown, Tom Wiggins, James B. Haddow, Atul Kapila, Dominic Coull, Andrew Walden

**Affiliations:** 1 Department of Surgery, Royal Berkshire NHS Foundation Trust, Reading, UK; 2 Intensive Care and Anaesthetics, Royal Berkshire NHS Foundation Trust, London Road, Reading, RG1 5AN UK; 3 London Surgical Research Group, Reading, UK

**Keywords:** General surgery, Ischemic preconditioning, Myocardial injury

## Abstract

**Background:**

Perioperative myocardial injury (PMI) is common in elective inpatient abdominal surgery and correlates with mortality risk. Simple measures for reducing PMI in this cohort are needed. This study evaluated whether remote ischemic preconditioning (RIPC) could reduce PMI in elective inpatient abdominal surgery.

**Methods:**

This was a double-blind, sham-controlled trial with 1:1 parallel randomization. PMI was defined as any post-operative serum troponin T (hs-TNT) > 14 ng/L. Eighty-four participants were randomized to receiving RIPC (5 min of upper arm ischemia followed by 5 min reperfusion, for three cycles) or a sham-treatment immediately prior to surgery. The primary outcome was mean peak post-operative troponin in patients with PMI, and secondary outcomes included mean hs-TnT at individual timepoints, post-operative hs-TnT area under the curve (AUC), cardiovascular events and mortality. Predictors of PMI were also collected. Follow up was to 1 year.

**Results:**

PMI was observed in 21% of participants. RIPC did not significantly influence the mean peak post-operative hs-TnT concentration in these patients (RIPC 25.65 ng/L [SD 9.33], sham-RIPC 23.91 [SD 13.2], mean difference 1.73 ng/L, 95% confidence interval − 9.7 to 13.1 ng/L, *P* = 0.753). The treatment did not influence any secondary outcome with the pre-determined definition of PMI. Redefining PMI as > 5 ng/L in line with recent data revealed a non-significant lower incidence in the RIPC cohort (68% vs 81%, *P* = 0.211), and significantly lower early hs-TnT release (12 h time-point, RIPC 5.5 ng/L [SD 5.5] vs sham 9.1 ng/L [SD 8.2], *P* = 0.03).

**Conclusions:**

RIPC did not at reduce the incidence or severity of PMI in these general surgical patients using pre-determined definitions. PMI is nonetheless common and effective cardioprotective strategies are required.

**Trial registration:**

This trial was registered with Clinicaltrials.gov, NCT01850927, 5th July 2013.

**Electronic supplementary material:**

The online version of this article (10.1186/s12871-018-0524-6) contains supplementary material, which is available to authorized users.

## Background

Perioperative myocardial injury (PMI) is defined as elevated cardiac enzymes after surgery with or without relevant symptoms or electrocardiogram (EKG) changes, and is proportionally linked to short-term mortality [1]. The incidence of PMI after inpatient abdominal surgery is common - 12-21% - and is higher still in selected subgroups (e.g. gastro-oesophageal resection > 50%) [1–3]. Cardioprotection studies in non-cardiac surgery have focused on clinical endpoints [4], using relatively hazardous interventions, such as medication or revascularization, in which harm outweighs benefit in lower risk patients [5–8]. However, given that PMI is also linked with adverse outcomes and is more common, new cardioprotection studies with biochemical endpoints and appropriately low-hazard interventions are needed.

Remote ischemic preconditioning (RIPC) is a simple and safe procedure that may reduce ischaemia-reperfusion injury [9, 10]. It involves temporary ischemia-and-reperfusion cycles to non-vital tissues, which may convey protective effects to distant sensitive organs. The specific mechanisms are incompletely understood, and may involve transcriptional reprogramming to cytoprotective cascades, and reduced radical release from the mitochondrial transition pore [11–13]. Harmful effects of RIPC seem limited to skin petechiae, which occur in 3–4% and are self-limiting [14, 15]. RIPC for cardioprotection has been investigated in cardiac [16], renal surgery [17], and vascular surgery [18], albeit with mixed results. However, whether RIPC influences PMI after abdominal surgery remains unknown.

We hypothesized that RIPC could reduce the frequency and severity of PMI after major abdominal surgery, and so we designed a sham-controlled randomized study to test this. The primary outcome measure was incidence of PMI (defined at our institution as > 14 ng/L) within 72 h of surgery, and secondary outcomes were high-sensitivity troponin T (hs-TNT) area under the curve (AUC), cardiovascular events and mortality.

## Methods

### Trial design

This was a single-centre, double-blind, sham-controlled trial with 1:1 parallel randomization. The patient, the clinical team, and the investigator analyzing the data were blinded to the provided treatment. The trial was performed between October 2013 and December 2015 at the Royal Berkshire Hospital, Reading, United Kingdom, a medium-sized district general hospital. Ethical approval for the study was obtained from the National Research Ethics Service Committee South Central (Berkshire, ref.: 13/SC/0306), and the study protocol was deposited with ClinicalTrials.gov (NCT01850927, released 5th July 2013). The CONSORT checklist was followed for reporting [19].

### Participants

Inclusion criteria were established as follows: (i) any patient undergoing a gastrointestinal or complex abdominal wall operation with expected admission > 1 night (ii) > 45 years of age. Exclusion criteria were patients (i) taking glibenclamide [20] (iii) with untreated hypertension (iii) with upper limb vascular disease or abnormal anatomy, including dialysis patients with arteriovenous fistulas (iv) unable to provide informed consent (v) participating in another cardiac trial (vi) recent diagnosis of infection (vii) elevated pre-operative hs-TnT. The consultant surgeon responsible for the patients care introduced the trial after confirmation of surgery. The research team took informed consent after the anesthetic pre-assessment visit, followed by baseline clinical and demographic data and a reference hs-TNT. Clinical cardiovascular risk factors were collected to predict PMI risk. The initial consideration was to develop the study as a pragmatic approach for clinical implementation, and so no restrictions were placed on the choice of surgical technique, and were at the discretion of the direct clinicians.

### Interventions

Pre-operative anesthesia was conducted in the anesthetic anteroom and was protocolized: (i) intravenous access (ii) spinal/epidural anesthesia provision (iii) general anesthetic induction following pre-oxygenation (iv) airway intubation (v) commence RIPC treatment (vi) intra-arterial and further intravenous access on contralateral arm (vii) urinary catheter (viii) central venous access (ix) finish RIPC treatment (x) move patient to theatre. The anethesiologist responsible for the case carried out induction, intubation and maintenance of anesthesia. Anesthesia was predominantly maintained using desflurane; some received a continuous propofol infusion. There is data suggesting cardioprotective benefit for volatile anesthesia [21], and abrogation of RIPC effect by propofol anaesthesia [22, 23], and further data showing cardioprotective equivalence in cardiac surgery [24]. Therefore, a post-hoc comparison of the two techniques was favoured, rather than pre-specification of maintenance.

The study treatments were carried out by the operational investigators (DC, NJ, AB). A dedicated and regularly calibrated study sphygmomanometer (Durashock DS54, Welch Allyn) was applied to an arm. For RIPC, a pressure of 200 mmHg was applied for 5 min, followed by 5 min rest, for three cycles, totalling a 30-min treatment. The cuff for non-invasive blood pressure monitoring was placed on a leg or on the RIPC arm after the 30-min treatment. Sham-RIPC was identical in all aspects except that the release valve on the sphygmomanometer was open throughout, so that cuff pressure was always < 15 mmHg. The clinical team, participants and investigators responsible for data analysis (SA, TW, AW) were thus blinded to allocation throughout the recruitment window.

### Outcomes

The primary outcome was the mean peak post-operative hs-TnT within 72 h in patients developing PMI, as the best available PMI data in abdominal surgery used this endpoint and was used to power the study [1]. PMI was defined as any post-operative hs-TnT > 14 ng/L (cut-offs based on the 99th percentile of the local population; at our Institution, a clinically significant value is > 14 ng/L [25]). Assays were measured under research contract with the Institution’s biochemistry department, using the 5th generation Elecsys system (Roche, Basel, Switzerland), complying to standard clinical-grade quality control and calibration procedures. Results were retained by the pathology department until the completion of recruitment and follow-up, and thus could not influence the usual clinical course. Blood samples were collected at 6 to 12 h, 24 h, 48 h, and 72 h. Secondary outcomes included mean hs-TnT at individual timepoints, hs-TNT area-under-the-curve within 72 h, length of hospital stay, significant surgical complications within 30 days, and major adverse clinical cardiovascular events and all-cause mortality within 1 year. Specific (skin petechiae) and general serious adverse events were collected. Definitions for all outcomes are provided in Additional file 1. Data were collected at day of consent, day of surgery, day of discharge, and one-year follow-up. These were all direct encounters except for follow-up (telephone call or direct).

### Sample size

Sample sizes were calculated using peak post-operative troponin data from the 2012 VISION paper and meta-analyzed estimates of RIPC efficacy in cardiac surgery [1, 26, 27], as no studies have assessed the use of RIPC in general surgery. We estimated mean peak hs-TnT rise in positive elective patients to be 30 ng/L, with a standard deviation of 6 ng/L. Meta-analyses at the time of study design reported an RIPC effect size of approximately 40% in patients undergoing cardiac surgery. With a two-tailed significance of 0.05, power 0.8, dropout rate of 5%, and a significant (i.e. ≥14 ng/L) hs-TnT rise occurring in 22% of patients this returns a recruitment target of 84.

### Randomization & blinding

Patients were randomized using a sealed envelope system. A non-operational co-author (TW) randomly inserted 50 each of treatment and sham designations into 100 sequentially number envelopes. The allocation sequence was generated using a truly random number generator (based on atmospheric noise, www.random.org). The envelope was opened after intubation by the operational investigator performing the treatment (DC, NJ, AB), and the treatment was recorded in the site file. This was a double-blind study in which the patient and the entire clinical team were blinded to the treatment assignment throughout. All analysis was undertaken blindly by a non-operational co-author (TW, treatment assignments recoded to “A” and “B”).

### Statistical methods

Continuous group characteristics were described by median and interquartile range and compared with Mann-Whitney U-test (MWU); categorical variables were described by frequency and groups compared with Fisher’s exact test. Peak and timepoint post-operative hs-TnT were compared with MWU, and hs-TnT curve integrals were compared using parametric tests. Analysis was by intention-to-treat. Predictors of PMI were discovered using chi-squared tests. Analysis was undertaken in SPSS (version 22, IBM, Armonk, NY, USA).

## Results

### Participants

**Fig. 1:**
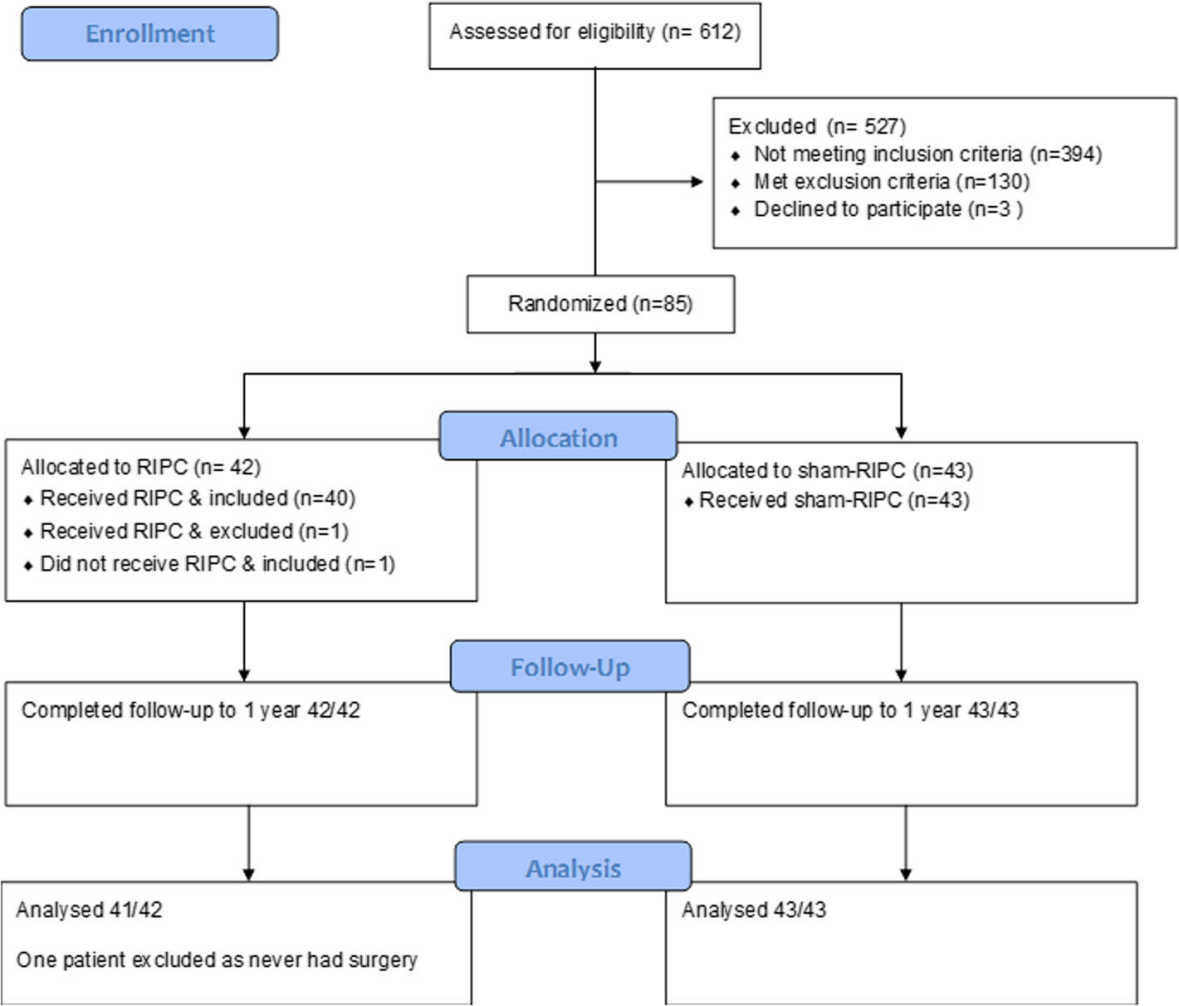
CONSORT flowchart of participant allocation and progress

Between October 2013 – December 2015, from a total of 612 screened general surgical patients, 85 were recruited to the study and randomized to RIPC (42) or sham-RIPC (43) (see Fig. 1). The main reason for exclusion was projected stay ≤1 night. One patient was withdrawn after randomization as she developed a severe bradycardia at induction prior to receiving the RIPC treatment, leading to cancellation of surgery and thus could not participate. Participants generally underwent major colorectal resections or large bowel anastomoses after emergency surgery (see Table 1). Other operations included splenectomy (2), sleeve gastrectomy (2), and complex abdominal wall reconstruction (1). There were no differences in baseline indices between the treatment arms (see Table 1). All 84 patients were successfully followed-up to 1 year.

**Table 1:** Participant demographics

Characteristic	Sham-RIPC (*n* = 43)	RIPC (*n* = 41)	*P* =
Age, years ^a^	60 (±15)	65 (±12)	0.261^†^
Male sex	25 (65%)	21 (51%)	0.271
BMI, kg/m^2 a^	25.5 (22.8, 31.1)	27.0 (22.5, 29.3)	0.589^‡^
Abnormal baseline ECG	9 (21%)	5 (12%)	0.385
Pre-op Creatinine (μmol/L) ^a^	81 (±20)	76 (±17)	0.431^†^
Cardiac RFs			
Hypercholesterolaemia	8 (19%)	6 (15%)	0.772
Hypertension	11 (26%)	11(27%)	1
FHx IHD	8 (19%)	8 (19%)	1
Ex/current smoking	29 (67%)	21 (49%)	0.182
Coronary Artery Disease	5 (12%)	1 (2%)	0.202
Diabetes	2 (5%)	3 (7%)	0.672
ASA			0.714
I	7 (16%)	5 (12%)	
II	29 (67%)	29 (71%)	
III	7 (16%)	6 (15%)	
IV	0	1 (2%)	
Maintenance anaesthesia			0.645
Propofol	3 (7%)	4 (10%)	
Desflurane	40 (93%)	37 (90%)	
Surgical procedure			0.857
Small Bowel	3 (7%)	5 (12%)	
Right hemicolectomy	12 (28%)	11 (27%)	
Left hemicolectomy	1 (2%)	0	
Rectum or sigmoid colon	21 (49%)	19 (46%)	
Colectomy/Proctocolectomy	3 (7%)	4 (10%)	
Other	3 (7%)	2 (5%)	

*BMI* body mass index, *ECG* electrocardiogram, *FHx IHD* family history of ischaemic heart disease

^a^Descriptives given as counts (percentages), except for continuous data, which is given as mean (± standard deviation), or median (25% percentile, 75% percentile)

*P*-values calculated with Fisher’s exact test, ^†^Student’s t-test, or ^‡^Mann-Whitney U-test

### Outcomes

**Fig. 2:**
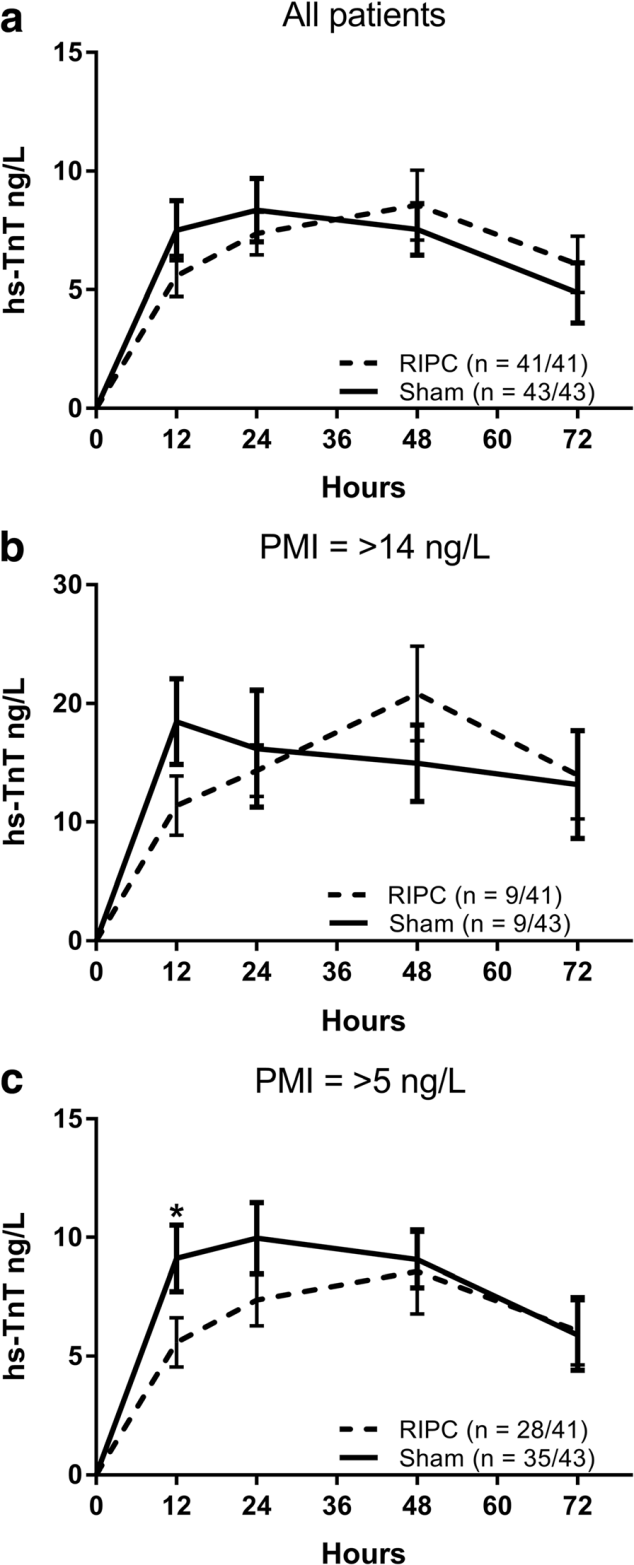
Mean post-operative high-sensitivity troponin T concentrations in the RIPC and sham-RIPC cohorts. **a** All patients included **b** Patients with PMI (any peak > 14 ng/L) **c** Patients with PMI (any peak > 5 ng/L). Error bars indicated standard deviation. *P < 0.05

A total of 394 hs-TnT values were collected, 3.7 post-operative results per patient. PMI was recorded in 9/41 patients (22%) in the RIPC group and 9/43 patients (21%) in the sham-RIPC group (*P* = 1.000) indicating that RIPC did not influence PMI incidence. The mean peak post-operative hs-TnT concentration in those with PMI in the RIPC cohort was 25.65 ng/L (standard deviation (SD) 9.33), and in the sham-RIPC cohort 23.91 ng/L (SD 13.2, mean difference 1.73 ng/L, 95% confidence interval − 9.7 to 13.1 ng/L, *P* = 0.753), indicating that RIPC did not affect PMI severity. The total mean hs-TnT area under the curve in those sustaining PMI was: RIPC 1.24 μg/L (SD 0.54 μg/L) vs sham-RIPC 0.97 μg/L (SD 0.35 μg/L, mean difference − 0.27 μg/L, 95% confidence interval − 0.92 to 0.39 μg/L, *P* = 0.393). There was no significant AUC mean difference at any individual timepoint either in all patients or the PMI group (see Fig. 2a and b), or in any secondary outcome (see Table 2). There were no reported adverse events attributable to the treatments. None of the seven patients who received propofol-based maintenance anesthesia developed a PMI, and so all patients who sustained PMI were maintained with desflurane.

**Table 2:** Study outcomes

	Sham-RIPC (*n* = 43)	RIPC (*n* = 41)	*P*
Peak hsTNT, n (%)			0.327
< 5 ng/L	8 (19%)	13 (31%)	
5–14 ng/L	26 (60%)	19 (46%)	
> 14 ng/L	9 (21%)	9 (22%)	
Mean peak hsTNT (ng/L)			
All	10.3 (5.6, 13.7)	7.8 (4.4, 12.9)	0.397
PMI (> 14 ng/L)	17.1 (16.5, 29.0)	20.5 (17.0, 35.0)	0.387
PMI (> 5 ng/L)	11.5 (8.8, 15.6)	7.7 (5.7, 11.0)	0.973
hsTnT AUC to 72 h			
All	394 (202, 527)	335 (130, 660)	0.700
PMI (> 14 ng/L)	868 (559, 1256)	816 (410, 1050)	0.711
PMI (> 5 ng/L)	481 (286, 669)	481 (320, 810)	0.548
Secondary outcomes			
MACCE to 1 year	4 (9%)	1 (2%)	0.36
Surgical complication	11 (26%)	8 (20%)	0.605
Length of stay	6 (4, 9)	5 (3, 8.5)	0.472
Sympathomimetic use	32 (80%)	27 (73%)	0.592
Venous lactate (mmol/L)	1.5 (1.0, 2.1)	1.2 (0.9, 1.75)	0.288
Venous creatinine (μmol/L)	78 (67, 96)	76 (64, 101)	0.666

Descriptives are count (percentage) and compared with chi squared tests, or median (25th percentile, 75th percentile) and compared with Mann-Whitney U test. *MACCE* major adverse cardiac and cerebrovascular events

Recent data suggests that hs-TnT values of > 5 ng/L after non-cardiac surgery are associated with 300% increase in mortality [3]. In light of this, hs-TnT curves were again assessed using this cut-off. It was found that the RIPC cohort had a non-significant lower incidence of PMI in the RIPC cohort (68% vs 81%, *P* = 0.211), and significantly lower early hs-TnT release (12 h time-point, RIPC 5.5 ng/L (SD 5.5) vs sham 9.1 ng/L (SD 8.2), *P* = 0.033, see Fig. 2c).

Associations between the pre-determined pre-operative characteristics and post-operative raised hs-TnT were also examined (see Table 3). On univariate analysis, increased hs-TnT was significantly associated with older patients, having > 3 cardiac risk factors, and an abnormal baseline EKG. Intra-operative raised venous lactate (> 2 mmol/L) was inversely associated with raised hs-TnT. Two patients were dead at 1 year, and both had post-operative hs-TnT values > 14 ng/L.

**Table 3:** Risk factors for elevated hs-TnT

	No PMI	PMI	*P* ^†^ *=*
Age > 70	17 (26%)	12 (63%)	0.005**
Male	35 (55%)	14 (74%)	0.187
BMI > 30	13 (23%)	5 (29%)	0.749
Cardiac RFs			
0	3 (5%)	0	1.000
1	35 (54%)	12 (63%)	0.423
2	17 (26%)	2 (10%)	0.339
3	7 (11%)	1 (5%)	1.000
> 3	3 (5%)	4 (21%)	0.035*
Pre-operative test			
Abnormal ECG	8 (13%)	6 (32%)	0.049*
Creatinine (> 120 μmol/L)	25 (39%)	3 (16%)	0.156
Operative events			
HR ×2 normal	4 (7%)	2 (12%)	0.616
MAP ×0.5 normal	8 (13.8%)	3 (16%)	1
Lactate > 1.5	14 (24%)	1 (5%)	0.098
Surgery > 4 h	32 (55%)	12 (63%)	0.602
Sympathomimetic use	43 (74%)	16 (84%)	0.535
Post-operative events			
Average length of stay (days)	8	8	1
Dead at 30 days	0	0	–
Dead at 1 year	0	2 (12%)	0.044*

^†^*P* values calculated with Fisher exact or Mann-Whitney U test

## Discussion

This was a randomized controlled trial designed to test whether RIPC can reduce PMI caused by elective major abdominal surgery. Within this cohort, RIPC was safe. Nearly a quarter of patients had a clinically significant PMI, supportive of several recent studies [1, 2, 28, 29]. However, RIPC did not affect PMI incidence or severity, or associated clinical outcomes. More recent outputs of the VISION collaboration suggests that absolute hs-TnT values of > 5 ng/L are associated with modest risk of mortality [3]. When the additional patients were included in the present analysis, RIPC significantly decreased the magnitude of hs-TnT at 12 h (*P* < 0.033).

Promising animal studies [30], human randomized controlled trials [16, 31, 32] and meta-analyses [26, 27] showed that RIPC can reduce the PMI associated with cardiac surgery. However, two meta-analyses have recently questioned whether RIPC affected clinical outcomes in this cohort [33, 34]. As the present study closed, two international RCTs (ERRICA and RIPHeart) powered to differences in detect a composite clinical outcome after cardiac surgery failed to show a treatment benefit for RIPC, both in terms of clinical and biochemical endpoints [14, 15]. The neutral findings of RIPC in the present study may be explained by (i) the pathophysiology of myocyte injury in this surgical context is not influenced by RIPC (ii) no pre-specification of anesthesia (iii) inadequate definition of PMI in this surgical context.

To our knowledge, this is the first randomized controlled trial to address whether RIPC can influence PMI in patients undergoing inpatient abdominal surgery. The study was limited by the lack of available data on treatment effects in this surgical setting, so surrogate data from cardiac surgery was used, adjusting for PMI incidence in general surgery using VISION data. The second potential limitation is that anesthesia was not pre-specified. There is limited evidence from dog and human studies that propofol maintenance abrogates the RIPC effect [23, 35], and it is interesting that the small group of propofol-only patients did not develop PMI. In 90% ERRICA and 100% RIPHeart participants, anesthesia was solely propofol-based. Advocates of RIPC have suggested that volatile anesthesia may yield different results [36, 37]. However, there was still no RIPC benefit in those with PMI (> 14 ng/L), who all received volatile anesthesia.

Several studies have found PMI to be common after inpatient abdominal procedure, and that it predicts mortality [1, 28, 38, 39]. Confounding influences such as infective respiratory disease, renal impairment, and concurrent use of diuretics and anticoagulants are not fully characterized [40]. In this study, pre-existing infection was an exclusion criterion, and the other factors were not associated with raised hs-TnT (see Table 3). In keeping with previous studies [38, 41], factors predictive of risk were age and abnormal pre-operative cardiac tests, suggesting genuine and presumably modifiable myocyte pathology. Thus, two lines of future investigation are proposed (i) perioperative studies using alternative cardioprotective therapies to reduce PMI (ii) post-operative therapeutic studies in which the presence of PMI stratifies to long-term risk reduction. Although RIPC may not be efficacious in treating PMI in the present general surgery study population, it may yet hold value in certain high-risk contexts, for example in oesophageal resections.

## Conclusion

RIPC was not effective in reducing PMI in this patient cohort. Perioperative myocardial injury in elective major abdominal surgery is common, and simple measures to provide cardioprotection are needed.

## Additional file

Additional file 1: Study definitions. (DOCX 34 kb)

## Abbreviations

AUC: area under the curve
EKG: Electrocardiogram
MWU: Mann-Whitney U-test
ns-TNT: high sensitivity troponin T
PMI: perioperative myocardial injury
RIPC: remote ischaemic preconditioning
SD: Standard deviation
