# Supplementary material for: Remote ischemic preconditioning for cardioprotection in elective inpatient abdominal surgery – a randomized controlled trial
Source: BMC Anesthesiol. 2018 Jun 26;18:76. doi: 10.1186/s12871-018-0524-6 (PMC6020340; doi:10.1186/s12871-018-0524-6)
Supplement: Supplementary file 1 — Study definitions. (DOCX 34 kb) [file 12871_2018_524_MOESM1_ESM.docx]

# Additional file 1: Study definitions

## Post-operative myocardial injury (PMI)

Any post-operative high-sensitivity troponin T value >14 ng/L

## Major Adverse Cardiovascular or Cerebrovascular Events

Any of:

1. Non-fatal cardiac arrest
2. Acute myocardial infarction
3. Angina
4. New cardiac arrhythmia
5. Congestive heart failure
6. Stroke
7. Cerebrovascular death
8. Cardiovascular death

## Non-fatal Cardiac Arrest

An absence of cardiac rhythm or presence of chaotic rhythm requiring any component of basic or advanced cardiac life support.

## Acute Myocardial Infarction

Increase and gradual decrease in troponin level in the company of at least one of the following: ischaemic symptoms, abnormal Q waves on the ECG, ST-segment elevation or depression; or coronary artery intervention (e.g. coronary angioplasty) or a typical decrease in an elevated troponin level detected at its peak after surgery in a patient without a documented alternative explanation for the troponin elevation

## Angina

Dull diffuse substernal chest discomfort precipitated by exertion or emotion and relieved by rest or glyceryl trinitrate.

## New Cardiac Arrhythmia

ECG evidence of atrial flutter, atrial fibrillation, or second- or third-degree atrioventricular conduction block

## Congestive Heart Failure

New in-hospital signs or symptoms of dyspnoea or fatigue, orthopnoea, paroxysmal nocturnal dyspnoea, increased jugular venous pressure, pulmonary rales on physical examination, cardiomegaly, or pulmonary vascular engorgement.

## Stroke

Embolic, thrombotic or haemorrhagic event lasting at least 30 min with or without persistent residual motor, sensory, or cognitive dysfunction; if the neurological symptoms continue for more than 24 h, a person is diagnosed with stroke, and if lasting less than 24 h the event is defined as a transient ischaemic attack.

## Cerebrovascular Death

A death caused by cerebrovascular disease

## Cardiovascular Death

Any death, unless an unequivocal non-cardiovascular cause could be established.

## 30 day significant surgical complication

Are any post-operative complications of Clavien-Dindo grade III-IV, i.e. those requiring radiological, endoscopic or operative intervention. Examples would include (but are not limited to):

- Anastomotic leak/dehiscence requiring surgical or radiological intervention
- Surgical space infection
- Wound infection or dehiscence
- Bleeding requiring revision surgery

## Clavien-Dindo Classification:

1. Any physiological deviation from normal post-operative care (allows use of antipyretics, analgesics, antiemetics, diuretics, fluids, electrolytes, physiotherapy)
2. Complication requiring other pharmacological management (includes blood transfusions and total peripheral nutrition)
3. Complications requiring interventional, non-operative management
4. Complication requiring intensive care
5. Death

## Sympathomimetic use

Any use of intra-operative sympathometic medication including metaraminol, ephedrine and noradrenaline

## 1.12 Abnormal ECG

Any non-sinus rhythm including any heart block, q wave, poor r wave progression, ST changes, and right or left bundle branch block.
